# Lin28 Induces Epithelial-to-Mesenchymal Transition and Stemness via Downregulation of Let-7a in Breast Cancer Cells

**DOI:** 10.1371/journal.pone.0083083

**Published:** 2013-12-11

**Authors:** Yujie Liu, Haiyan Li, Juan Feng, Xiuying Cui, Wei Huang, Yudong Li, Fengxi Su, Qiang Liu, Jiujun Zhu, Xiaobin Lv, Jianing Chen, Di Huang, Fengyan Yu

**Affiliations:** 1 Department of Breast Surgery, Sun Yat-Sen Memorial Hospital, Sun Yat-Sen University, Guangzhou, People’s Republic of China; 2 Key Laboratory of Malignant Tumor Gene Regulation and Target Therapy of Guangdong Higher Education Institutes, Sun Yat-Sen Memorial Hospital, Sun Yat-Sen University, Guangzhou, China; 3 Department of Breast and Thyroid Surgery, The 6^th^ Affiliated Hospital of Sun Yat-Sen University, Guangzhou, People’s Republic of China; 4 Department of Xiangyang Hospital, Hubei University of Medicine, Xiangyang, People’s Republic of China; 5 Center for Medical Research, Sun Yat-Sen Memorial Hospital, Sun Yat-Sen University, Guangzhou, People's Republic of China; Wayne State University School of Medicine, United States of America

## Abstract

The RNA-binding protein Lin28 is known to promote malignancy by inhibiting the biogenesis of let-7, which functions as a tumor suppressor. However, the role of the Lin28/let-7 axis in the epithelial-to-mesenchymal transition (EMT) and stemness in breast cancer has not been clearly expatiated. In our previous study, we demonstrated that let-7 regulates self-renewal and tumorigenicity of breast cancer stem cells. In the present study, we demonstrated that Lin28 was highly expressed in mesenchymal (M) type cells (MDA-MB-231 and SK-3rd), but it was barely detectable in epithelial (E) type cells (MCF-7 and BT-474). Lin28 remarkably induced the EMT, increased a higher mammosphere formation rate and ALDH activity and subsequently promoted colony formation, as well as adhesion and migration in breast cancer cells. Furthermore, we demonstrated that Lin28 induced EMT in breast cancer cells via downregulation of let-7a. Strikingly, Lin28 overexpression was found in breast cancers that had undergone metastasis and was strongly predictive of poor prognoses in breast cancers. Given that Lin28 induced the EMT via let-7a and promoted breast cancer metastasis, Lin28 may be a therapeutic target for the eradication of breast cancer metastasis.

## Introduction

Lin28 is a highly conserved RNA-binding protein that was initially identified as an important regulator of developmental timing in Caenorhabditis elegans [1]. The human Lin28 family is composed of two homologs: Lin28 (also known as Lin28a) and Lin28b. Lin28 is specifically expressed in undifferentiated embryonic stem cells (ESCs). However, Lin28 expression is dramatically downregulated in most normal adult tissues [2]. Ectopic expression of Lin28 has been observed in a wide range of tumors with advanced stage, including hepatocarcinomas, lung cancers, ovarian carcinomas, colon adenocarcinomas, and chronic myeloid leukemia [3–6]. Furthermore, Lin28 overexpression has been found to be a powerful predictor of poor prognosis and is adversely correlated with clinical outcomes and patient survival from primary breast tumors [7,8].

One of the downstream targets of Lin28 is let-7, which has been widely studied to function as a tumor suppressor by regulating multiple oncogenic signaling pathways. Recently, Lin28 was reported to regulate glucose metabolism via let-7 [9,10]. Lin28 can bind to the terminal loops of pre-let-7 elements and induce terminal uridylation of let-7 precursor microRNA, thus blocking their processing into mature miRNAs [11]. Decreased let-7 expression has been linked to increased tumorigenicity and poor patient prognosis in several cancers, including lung cancer, colorectal cancer, hepatic cancer, head and neck squamous cell carcinomas, and breast cancer [12–15]. Further research demonstrated that let-7 functions as a novel regulator of the epithelial-to-mesenchymal transition (EMT), facilitating tissue remodeling from the epithelial phenotype to mesenchymal phenotype, and is considered to be a prerequisite for tumor infiltration and metastasis [16–18]. Knockdown of let-7 significantly promotes EMT traits, whereas overexpression of let-7 efficiently reverses the EMT phenotype in oral and pancreatic cancer cells [16,19]. Downregulation of let-7 levels initiates and maintains oncostatin M-induced EMT via high-mobility group A protein 2 in breast cancer cells [20]. Moreover, our previous study and other reports had demonstrated that let-7 repression was largely responsible for cancer stemness and regulated stem cell differentiation and self-renewal capacity [21,22]. As one of the stem cell factors, Lin28 together with OCT4, SOX2, and NANOG can promote the reprogramming of a terminally differentiated cell to an induced pluripotent stem cell, which has been linked to oncogenesis [23].

In the present study, by overexpressing and suppressing Lin28, we demonstrated that Lin28 remarkably induced EMT and promoted adhesion and migration in breast cancer cells. Furthermore, we found that Lin28 induced the EMT in breast cancer cells through the repression of let-7a, and Lin28 overexpression was strongly predictive of poor prognosis in breast cancers.

## Materials and Methods

### Cell lines and culture

MCF-7, MDA-MB-231 and BT474 cell lines were purchased from American Type Culture Collection (ATCC, Manassas, VA, USA). The SK-3rd cell line used in this study was previously established by consecutively passaging the SKBR3 breast cancer cell line in non-obese, diabetic, severe-combined immunodeﬁcient mice under the pressure of chemotherapy [23]. Cells were cultured in Dulbecco’s Modified Eagle’s Medium (DMEM; Invitrogen, Carlsbad, CA, USA) supplemented with 10% fetal bovine serum (FBS; HyClone, South Logan, UT, USA). All of the cell lines were maintained in a humidified atmosphere containing 5% CO_2_.

### Mammosphere Culture

Mammosphere culture was performed as previously reported [22]. Cells (1000 cells/mL) were cultured in suspension in serum-free DMEM-F12 (Invitrogen, USA) supplemented with B27 (1:50, Invitrogen, USA), 20 ng/mL EGF (BD Biosciences, USA), 0.4% bovine serum albumin (Sigma, USA), and 4 mg/mL insulin (Sigma, USA).

### RNA oligoribonucleotides and plasmids

The Lin28 open reading frame was cloned into the pcDNA3.1(+) vector (Invitrogen) to express Lin28 (pc-Lin28) in human cells. The empty pcDNA3.1(+) vector (vec) was used as a control. Mutagenesis of Lin28 (Lin28-mut) was carried out using a site-directed mutagenesis kit (Stratagene, USA) to generate the Lin28 CCHC mutant, as previously described [11]. Let-7 mimics and negative control (NC) mimics were purchased from RiboBio (Guangzhou, CH). All siRNA oligoribonucleotides were purchased from GenePharma (Shanghai, CH). The sense and anti-sense strands of the siRNAs are shown in Table 1.

**Table 1:** The sense and anti-sense sequences of the siRNAs.

siRNA	Sense	Antisense
Lin28 siRNA-1	CAGUGGAGUUCACCUUUAATT	UUAAAGGUGAACUCCACUGTT
Lin28 siRNA-2	CUGGUGGAGUAUUCUGUAUTT	AUACAGAAUACUCCACCAGTT
GFP-siRNA	UAGCGACUAAACACAUCAATT	UUGAUGUGUUUAGUCGCUATT

### Cell transfection

Breast cancer cells were transiently transfected with GFP-siRNA, Lin28-siRNAs, let-7a mimics, NC mimics, vec, pc-Lin28, or the Lin28-mut plasmid using Lipofectamine 2000 (Invitrogen) according to the manufacturer's protocol. For the stable transfections, MCF-7 cells were exposed to 1000 ng per ml of G418 24 hrs after transfection with pc-Lin28, Lin28-mut or vec. G418-resistant cells were further cultured to select two Lin28 mRNA and protein expressing monoclones (pc-Lin28-1 and pc-Lin28-2).

### Western blotting

Protein extracts were resolved by 10% SDS-PAGE electrophoresis (BioRad, Berkeley, CA, USA), transferred to PVDF membranes (Roche, USA), and probed with antibodies against human Lin28a (1:1000, CST, USA), E-cadherin (1:1000, CST, USA), vimentin (1:1000, R&D, USA), or GAPDH (1:10000, Sigma, USA). Peroxidase-conjugated anti-rat or rabbit IgG (1:3000, CST, USA) were used as the secondary antibodies, and the antigen-antibody reactions were visualized using enhanced chemiluminescence (Thermo, Rockford, USA).

### PCR and Quantitative real-time PCR (qRT-PCR)

The total RNA was extracted using TRIzol (Invitrogen) according to the manufacturer’s instructions. Further miRNA reverse transcription was performed using a miRNA reverse transcription kit (Takara, Dalian, CH). Lin28 PCR amplification was performed with a gel imaging system (Bio-Rad) using a Premix ExTaq kit (Takara, Dalian, CH), and real-time PCR was performed with a Light Cycler480 system (Roche Diagnostics, Switzerland) using a SYBR Premix ExTaq kit (Takara, Dalian, CH). The relative amounts of the target miRNA were normalized to those of U6. The oligonucleotide sequences of the PCR and qRT-PCR primers are listed in Table 2.

**Table 2:** Oligonucleotide sequences of the PCR and qRT-PCR primers.

Gene	Forward primer	Reverse primer
Lin28a	TTGTCTTCTACCCTGCCCTCT	GAACAAGGGATGGAGGGTTTT
GAPDH	ATCACCATCTTCCAGGAGCGA	CCTTCTCCATGGTGGTGAAGAC
Let-7a	GGTGAGGTAGTAGGTTGTATAGTT	Uni-miR qPCR primer (TaKaRa)
U6	ACGCAAATTCGTGAAGCGTT	Uni-miR qPCR primer (TaKaRa)

### Flow cytometry analysis (FCM)

Samples were run on a Becton Dickinson Coulter flow cytometer. Mammospheres, either from the stably transfected MCF-7 pc-Lin28-1 and pc-Lin28-2 cells or after 3 cycles of siRNA transfection of MDA-MB-231 cells, were harvested by gentle trypsinization. Flow cytometry analysis of the cell markers was carried out using the Enzymatic Aldefluor Assay, which was used to test ALDH1 enzymatic activity following the manufacturer’s instruction.

### Soft agar assay

Mixtures (1.5 to 2 ml) of 2×DMEM/F12 and 1.36% low melting point agarose (Invitrogen) were added to 6-well plates to generate the bottom layer. Subsequently, 1.0 milliliter of 0.66% low melting point agarose was mixed with the same volume of single-cell suspension, and the mixture was added onto the bottom layer. After shaking, the plate was cultured in the incubator at 37°C for 1-2 weeks. Fresh medium was added every 3-4 days, after which point the mammospheres were counted and photographed under phase-contrast microscopy (Nikon, Japan).

### Immunofluorescence

For immunofluorescent (IF) staining, the cells were incubated with primary antibodies against E-cadherin (1:100, CST), vimentin (1:50, R&D), Lin28a (1:100, CST), or ALDH1 (1:100, R&D), followed by incubation with Alexa488/594-conjugated secondary antibodies (Invitrogen). Cell nuclei were counterstained with DAPI and imaged by confocal laser-scanning microscopy (Zeiss LSM710, Germany).

### Immunohistochemistry

Immunohistochemistry (IHC) was performed on paraffin-embedded breast cancer tissue sections using mouse monoclonal antibodies against Lin28a (1:100; CST). For the negative controls, isotype-matched antibodies were applied. Tissue sections were observed under a ZEISS AX10-Imager A1, and all pictures were captured using AxioVision 4.7 microscopy software.

### Adherence assay

The cells were suspended in serum-free medium and allowed to adhere to the plates, which had been pre-coated overnight with fibronectin (Roche, USA) followed by a 1-hr incubation with 10% BSA for 10 min. Next, the non-adherent cells were removed by gently washing the plates three times with PBS. The adherent cells were stained with crystal violet (0.005%, Sigma, USA) and counted as the number of cells per field of view using phase-contrast microscopy.

### Cell migration assay

Breast cancer cells (10^5^ cells/well) were plated on the upper inserts of the 24-well Boyden chambers (Corning, New York, NY, USA) with 0.2% BSA in serum-free DMEM media. After 8 hrs (MCF-7 cells) or 6 hrs (MDA-MB-231 cells) of culture at 37°C, the migrated cells that had crossed the inserts were stained with crystal violet (0.005%, Sigma) and were counted as the number of cells per field of view using phase-contrast microscopy. 

### siRNA-resistant Lin28 expression pcDNA 3.1 plasmid

Mutagenesis of Lin28 was carried out as previous described to generate the siRNA-insensitive Lin28 mutant (Lin28-resmut). There are total six point mutations within two Lin28-siRNA targeting sequences which didn’t alter the Lin28 amino acid sequence. Cotransfection of Lin28 siRNAs and siRNA–resistant Lin28 expression plasmid was performed by using Lipofectamine 2000 (Invitrogen) according to the manufacturer’s instructions. Following incubation for 6 hours, the medium was replaced with DMEM containing 10% FBS prior to further study. 

### Taqman qPCR assay for human let-7

Real-time quantitative PCR for mature human let-7 family members was performed using Applied Biosystems® Taqman® MicroRNA Assay (life technologies, Invitrogen, Carlsbad, CA, USA ) on a Light Cycler480 system (Roche Diagnostics, Switzerland). The assay ID were showed as below: let-7a (assay ID 000377), let-7b (assay ID 002619), let-7c (assay ID 000379), let-7d (assay ID 002283), let-7e (assay ID 002406), let-7f (assay ID 000382), let-7g (assay ID 002282), let-7i (assay ID 002221), miR-98 (assay ID 000577), miR-202 (assay ID 002363), miR202* (assay ID 002362) and U6 snRNA (assay ID 001973) as an endogenous control. All reactions were performed in a 20 μL reaction volume in triplicate according to the manufacture’s protocol, and the geometric average Ct value was used to calculate relative expression for each datapoint.

### Ethics Statement

The use of tissues for this study has been approved by the Ethics Committee of Sun Yat-Sen Memorial Hospital, Sun Yat-Sen University. The Ethics Committee of our hospital waived the need for written informed consent.

### Patient and Tissue Samples

Paraffin-embedded tumor tissues were obtained from 86 female breast cancer patients (median age 46 years, range 25-77) who were diagnosed at the Sun Yat-Sen Memorial Hospital, Sun Yat-Sen University, from January 2003 to December 2008. Pathological diagnosis, as well as ER and Her2 status, was veriﬁed by two different pathologists. Patients with breast tumors underwent comprehensive therapy according to the National Comprehensive Cancer Network (NCCN) guidelines. 

### Statistical analyses

All statistical analyses were performed using SPSS. Student's t-test was used to analyze the relationship between cell counts in the presence and absence of the vector or RNAi. All cell culture experiments were performed independently a minimum of three times. *P* values < 0.05 were considered to be statistically signiﬁcant in all cases. 

## Results

### Lin28 induced epithelial-to-mesenchymal transition in MCF-7 cells

**Figure 1:**
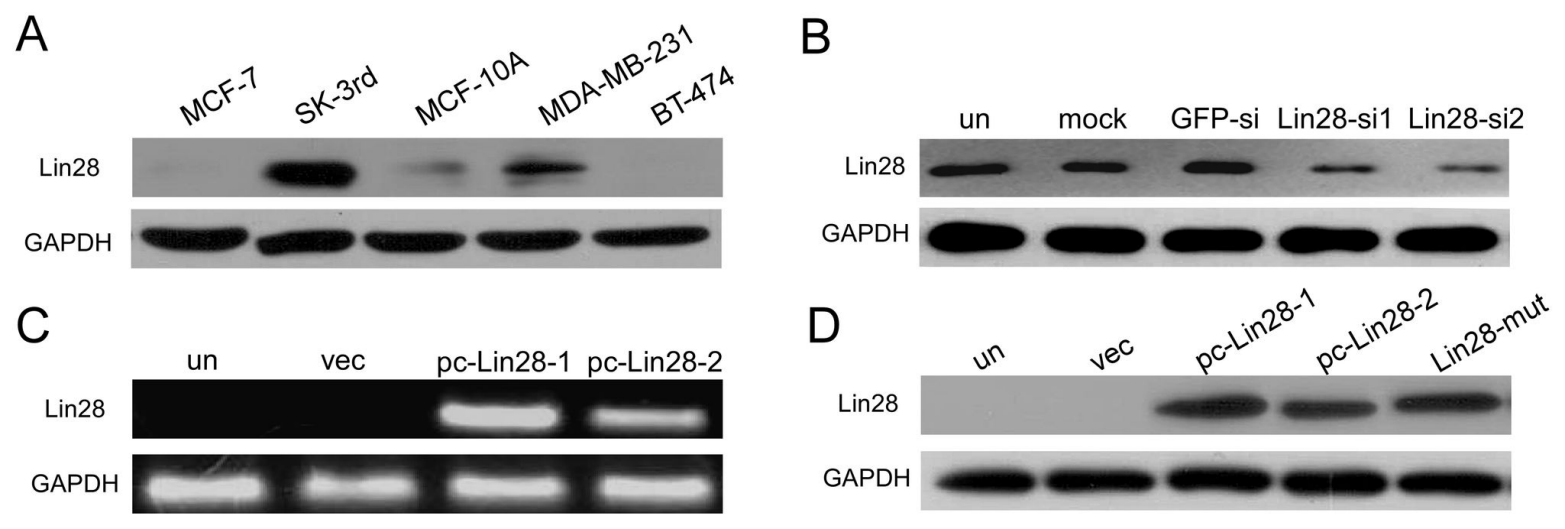
Lin28 expression in breast cancer cell lines and the transfection efficiencies of the Lin28-siRNAs, Lin28-expressing plasmid and Lin28-mutant plasmid. A. Differential expression of Lin28 was analyzed by Western blotting in multiple breast cell lines, including MCF-7, SK-3rd, MCF-10A, MDA-MB-231 and BT-474. B. Western blotting showed that transient transfection of either of the Lin28-siRNAs in MDA-MB-231 cells, compared with GFP-siRNA transfection, led to a reduction in the expression of Lin28. C and D. Lin28 expression was determined by PCR (C) and Western blotting (D) in MCF-7 cells that were stably transfected with pc-Lin28-1, pc-Lin28-2 (C and D) or Lin28-mut (D). GAPDH was used as a loading control.

Developmental research has shown that Lin28 expression is highly restricted to embryonic stem cells and that Lin28 is expressed at low levels in normal adult tissues [24]. In human tumors, Lin28 was up-regulated and functioned as an oncogene to promote malignant transformation and tumor progression [3]. In breast cancer cell lines, Lin28 was highly expressed in the mesenchymal (M) type MDA-MB-231 and SK-3rd cells, but it was barely detectable in the epithelial (E) type MCF-7 and BT-474 cells (Figure 1A). However, the expression pattern of Lin28b in breast cancer cells did not show an obvious trend (Figure S1).

To further investigate the function of Lin28 in breast cancer cells, we employed two Lin28-siRNAs that efficiently suppressed Lin28 expression in MDA-MB-231 cells (Figure 1B). In addition, we established two stable expression clones of Lin28 (pc-Lin28-1 and pc-Lin28-2) in MCF-7 cells, as well as a functionally dead Lin28 CCHC mutant (Lin28-mut), which failed to downregulate let-7 expression, as had been previously reported [11]. As shown in Figure 1C, pc-Lin28-1 and pc-Lin28-2 had increased levels of Lin28 mRNA expression when compared to their parental cells or MCF-7 cells that had been transfected with empty vector (vec). Stable protein expression of Lin28 and Lin28-mut in MCF-7 cells was detected by Western blotting (Figure 1D).

**Figure 2:**
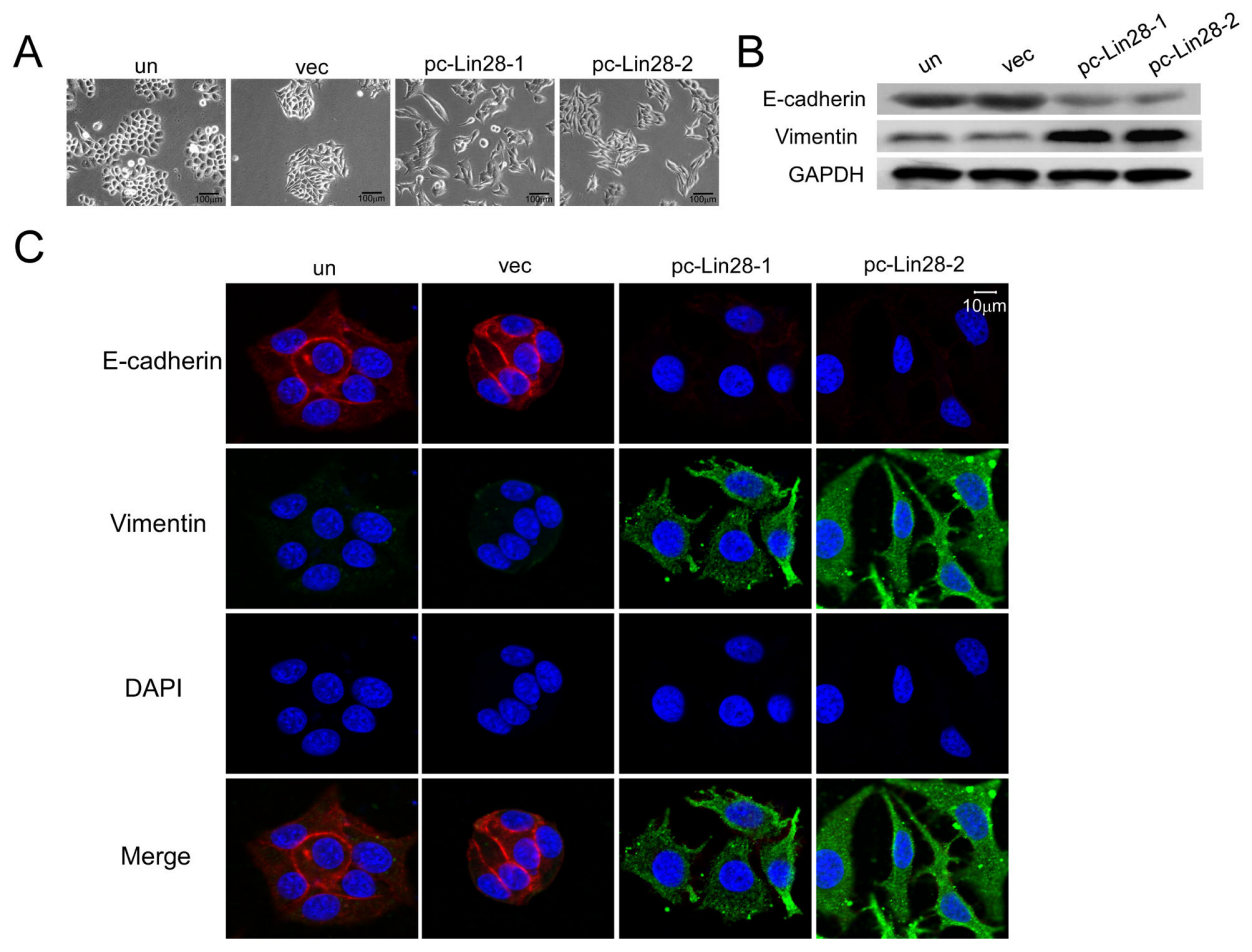
Lin28 promotes epithelial-to-mesenchymal transition in MCF-7 cells. A. Ectopic expression of Lin28 in pc-Lin28-1 and pc-Lin28-2 MCF-7 clones induced MCF-7 cells to adopt mesenchymal shapes. B and C. Western blotting (B) and Immunoﬂuorescence staining (C) illustrated reduced expression of E-cadherin and increased expression of vimentin in pc-Lin28-1 and pc-Lin28-2 cells. E-cadherin: red, vimentin: green, cell nuclei: blue. Bar, 10 μm.

Because up-regulation of let-7 led to the reversal of EMT in pancreatic cancer cells [19] and Lin28 downregulated let-7 expression, we analyzed whether Lin28 could induce the EMT in breast cancer cells. Cells stably expressing Lin28 displayed an obvious spindle shape and were separated from one another, whereas the parent cells and vector-transfected MCF-7 cells grew in clusters and were round with tight cell-cell junctions (Figure 2A). Furthermore, Western blotting and immunofluorescence staining demonstrated that the expression of the mesenchymal marker vimentin was dramatically increased. Concomitantly, the expression of the epithelial marker E-cadherin was decreased in pc-Lin28-1 and pc-Lin28-2 cells (Figure 2B and 2C), suggesting that Lin28 induced the EMT in breast cancer cells.

### Lin28 promoted adherence and migration in breast cancer cells

**Figure 3:**
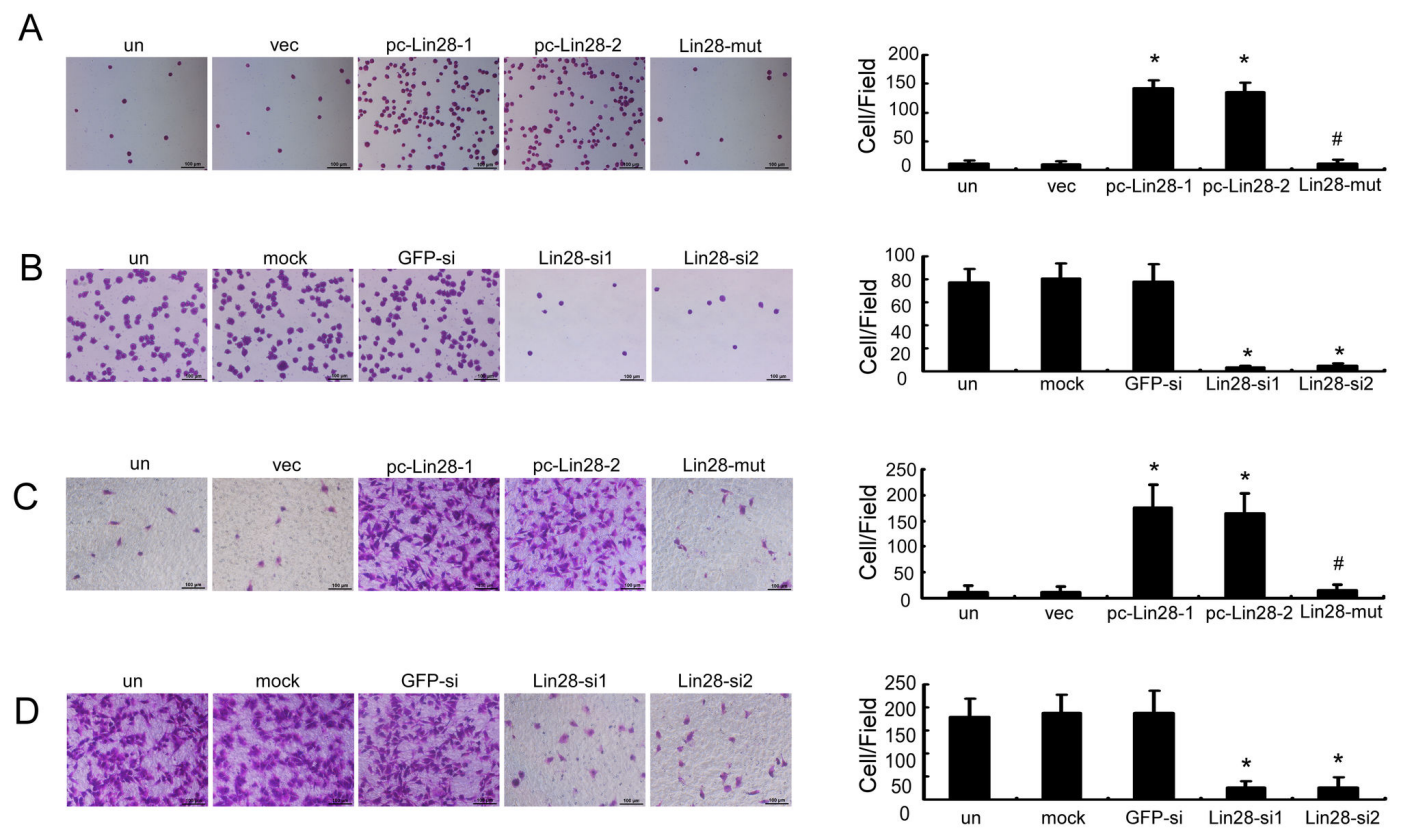
Lin28 promotes adhesion and migration in breast cancer cells. A and B. Cell adherence assays revealed that Lin28 stably expressed MCF-7 clones pc-Lin28-1 and pc-Lin28-2 adhered better to the ﬁbronectin-coated surface than empty vector and Lin28-mut transfectants (A). Meanwhile, cell adherence was remarkably decreased in MDA-MB-231 cells that had been transfected with Lin28-siRNA (B). C and D. Using Boyden chambers, cell migration was significantly increased in pc-Lin28-1 and pc-Lin28-2 MCF-7 cells (C), whereas it was significantly reduced in MDA-MB-231 cells after transfection with Lin28-siRNA (D). Error bars correspond to the mean ± SD. *P < 0.01as compared to the vec or GFPsi treatment group. Bars, 100 μm.

Because Lin28 can induce the EMT, which is often considered to be a prerequisite for tumor infiltration and metastasis, we further investigated the biological effects of Lin28 on breast cancer cells. Forced expression of Lin28 in MCF-7 cells (pc-Lin28-1 and pc-Lin28-2 cells) remarkably increased the number of cancer cells that adhered to a ﬁbronectin-coated surface by 13- and 12-fold as compared with vec MCF-7 cells, respectively (Figure 3A, p < 0.01), whereas the functionally dead Lin28 mutant had decreased numbers of adherent cells at levels that were comparable to those observed in the parent cells or vec MCF-7 cells (p > 0.05). We further found that transfection with either of the two Lin28-siRNAs efficiently inhibited the adherence of MDA-MB-231 cells to fibronectin by 17- and 16-fold, respectively, when compared to the cells submitted to GFP-siRNA transfection or mock transfection (Figure 3B, p < 0.01). 

Similar results were obtained in the migration assay: pc-Lin28-1 and pc-Lin28-2 cells demonstrated 16- and 14-fold increases in the number of migrated cells, whereas the Lin28-mut had no effect on migration when compared to vec MCF-7 cells (Figure 3C, p < 0.01). In addition, the migration of Lin28 knockdown MDA-MB-231 cells was downregulated by 7- and 6-fold when compared to the mock transfection or GFP-siRNA transfection (Figure 3D, P < 0.01). Taken together, our observations indicated that Lin28 played an important role in the invasive capacity of breast cancer cell lines in vitro.

### Lin28 promoted EMT via downregulation of let-7a in breast cancer cells

**Figure 4:**
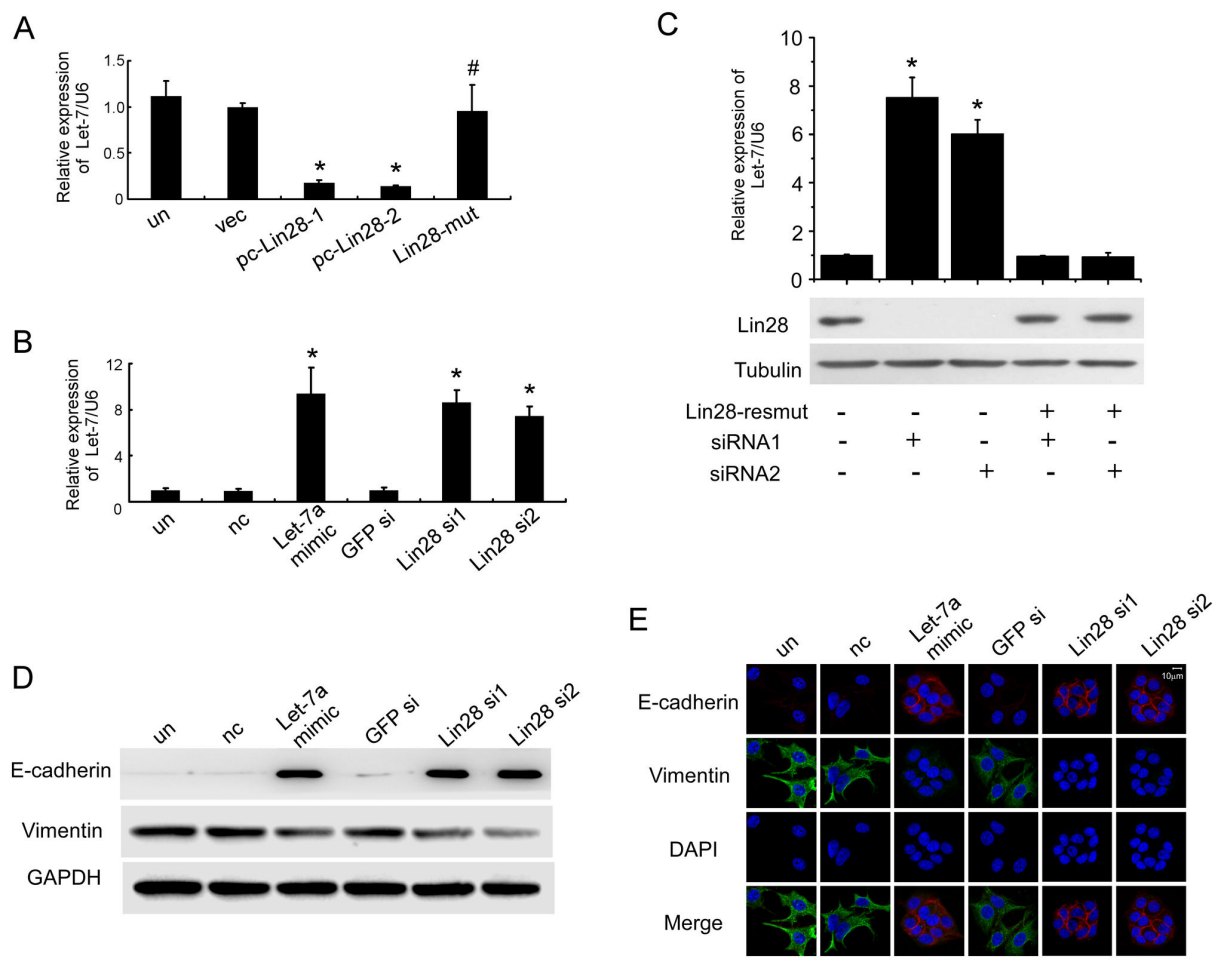
Lin28 induces EMT by inhibiting let-7a expression. The expression of let-7a, normalized to U6, was decreased in pc-Lin28-1 and pc-Lin28-2 MCF-7 cells when compared to empty vector and Lin28-mut transfectants (A), and the levels of let-7a were restored by transfection of let-7a mimics or Lin28-siRNA in pc-Lin28-1 (B). (C) Quantitative RT-PCR to assess Let-7a expression in pc-Lin28-1 cells cotransfected with Lin28-resmut and Lin28 siRNAs using Lipofectamine 2000. U6 was used as a loading control. Western blotting (D) and Immunoﬂuorescence staining (E) illustrated increased expression of E-cadherin and reduced expression of vimentin in pc-Lin28-1 cells that had been transfected with let-7a mimics and Lin28-siRNA. E-cadherin: red, vimentin: green, cell nuclei: blue. Bar, 10 μm.

Lin28 has been shown to block the biogenesis of let-7, which has been proven to play an essential role in regulating stem cell self-renewal, differentiation and the reversal of EMT. Lin28 overexpression considerably inhibited let-7 mRNA levels in breast cancer cells [7]. We used a Taqman qPCR miRNA assay to detect the expression of 11 members of the Let-7 family in pc-Lin28-1, pc-Lin28-2 and MCF-7 cells that were transfected with empty vector (vec). We found that overexpression of Lin28 could inhibit the expression of all Let-7 members (Figure S2). Among them, Let-7a was highly expressed in vec MCF-7 cells, and stable expression of Lin28 caused an approximate reduction of 88.9%±2.3% in pc-Lin28-1 and 57.4%±2.1% in pc-Lin28-2 (Figure 4A, Figure S2). Because Let-7a was abundantly expressed in vec MCF-7 cells and dramatically regulated by Lin28, we studied the function of Let-7a in this study. 

To further determine the role of let-7a in Lin28-induced EMT, we employed let-7a mimics and Lin28-siRNAs to restore let-7a expression. Figure 4B showed that stably expressing pc-Lin28-1 cells that had been transfected with either let-7a mimics or Lin28-siRNAs displayed dramatic restorations of let-7a expression levels, whereas transfection of the negative control mimics and GFP-siRNA did not affect let-7a expression. To verify that the recovery of let-7a in pc-Lin28-1 cells transfected with Lin28-siRNAs was mediated by knocking down Lin28, we constructed the siRNA-resistant Lin28 mutant vector (Lin28-resmut), which has six conservative point mutations within the Lin28-siRNAs targeting sequences that did not alter the Lin28 amino acid sequence (Figure S3). Co-transfection of Lin28-siRNAs with the Lin28-resmut in pc-Lin28-1 cells completely reversed the effects of Lin28-siRNAs by decreasing the expression of the let-7a mRNA and increasing the expression of the Lin28 protein to the level of the mock group (Figure 4C). These results suggested that the restoration effects of the siRNAs were consequences of silencing Lin28. In pc-Lin28-1 cells, Western blotting and immunofluorescence staining demonstrated that let-7a mimics and Lin28-siRNAs could increase the expression levels of E-cadherin and decrease those of vimentin, whereas transfection with either the negative control mimics or GFP-siRNA did not affect E-cadherin or vimentin expression levels (Figures 4D and E). These results illustrated the fact that Lin28-induced EMT in breast cancer cells is dependent upon reductions of let-7a.

### Lin28 increased mammosphere formation rate and ALDH activity in breast cancer cells

**Figure 5:**
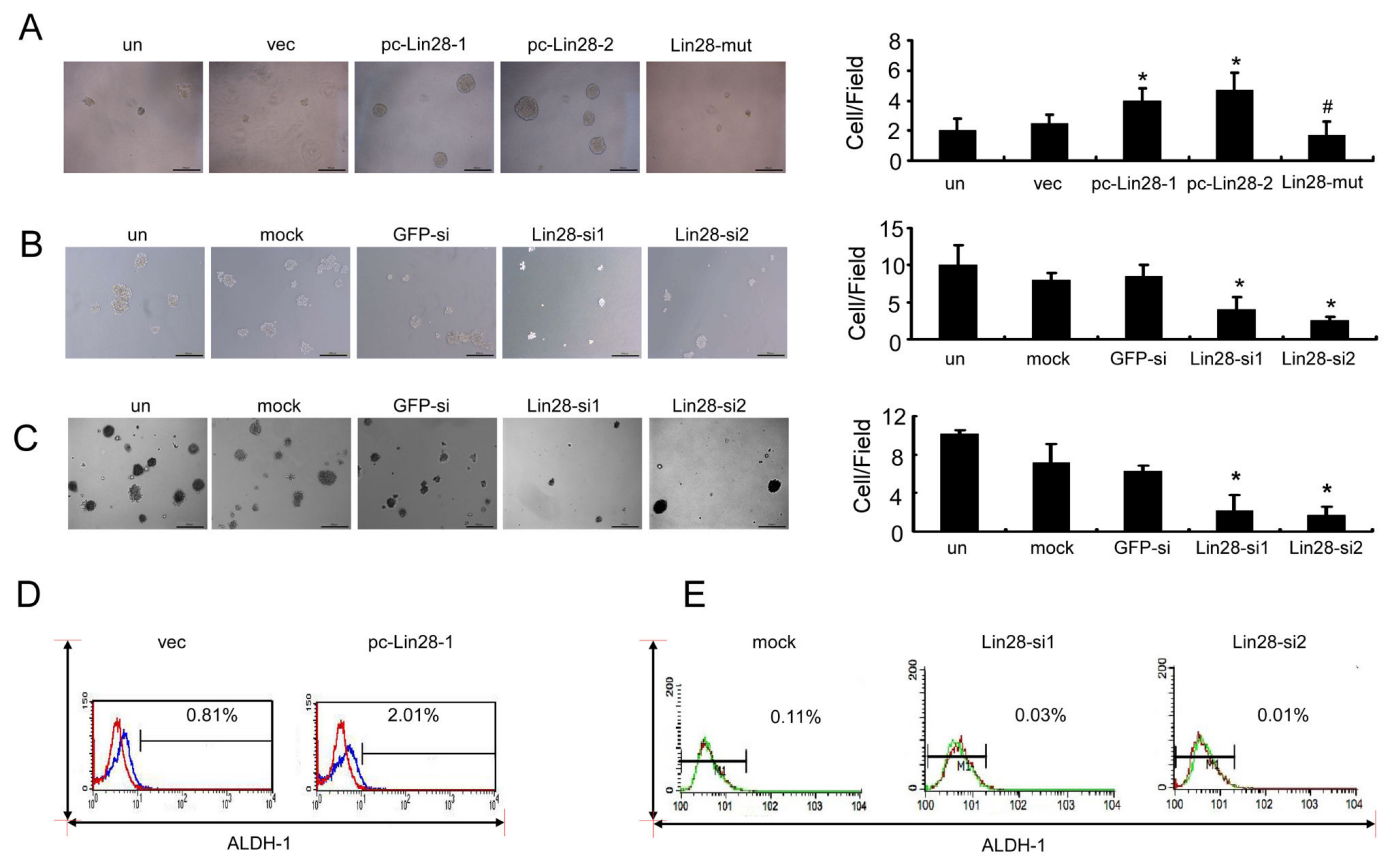
Lin28 increased the mammosphere formation rate and ALDH1 activity in breast cancer cells. A and B. Sphere-forming assay revealed that the Lin28 stably expressed MCF-7 clones pc-Lin28-1 and pc-Lin28-2 displayed an increased mammosphere formation rate when compared with empty vector and Lin28-mut transfectants (A). Meanwhile, the mammashpere formation rate was remarkably decreased in MDA-MB-231 cells that had been transfected with Lin28-siRNA (B). C. A soft-agar assay revealed that colony formation decreased in MDA-MB-231 cells after transfection with Lin28-siRNAs. D and E. By FCM, pc-Lin28-1 cells showed a 3-fold increase of ALDH1 activity as compared with empty vector cells (D). The proportion of ALDH1-positive cells was remarkably decreased in MDA-MB-231 cells that had been transfected with Lin28-siRNA (E). Error bars correspond to the mean ± SD. *P < 0.01 as compared to the vec or GFPsi treatment cells. Bars, 500 μm.

Recently, Lin28 was proven to facilitate the reprogramming of human somatic cells to induced pluripotent stem cells [25]. To examine whether Lin28 increased the stemness of MCF-7 cells, we compared the proportion of in vitro self-renewal cancer cells with pc-Lin28-1 and pc-Lin28-2 to parental MCF-7 and vec MCF-7 cells. By the mammosphere formation assay, a method for culturing mammary gland progenitor cells and breast cancer stem cells, we found that tumor cells with pc-Lin28-1 and pc-Lin28-2 showed a higher mammosphere formation rate, whereas the Lin28-mut mammosphere formation rate was decreased to a level that was comparable to those observed in the parent cells or vec MCF-7 cells (Figure 5A). Conversely, in MDA-MB-231 cells that had higher Lin28 expression, tumorsphere formation was remarkably disrupted by 2.5-fold and 4-fold after transfection with Lin28-si1 and Lin28-si2, respectively, when compared to transfection with GFP-siRNA (Figure 5B). Moreover, colony formation in soft agar was reduced by nearly 5-fold and 6-fold in MDA-MB-231 cells transfected with Lin28-si1 and Lin28-si2, respectively, when compared with GFP-siRNA-treated cells (Figure 5C, p < 0.01).

It has been shown that normal and malignant human mammary epithelial cells with higher aldehyde dehydrogenase activity (ALDH1) have stem/progenitor properties [26]. By using the Enzymatic Aldefluor assay, we found a higher ALDH1 activity in pc-Lin28-1 MCF-7 cells than empty vector-transfected cells (a 3-fold increase) (Figure 5D). On the contrary, in MDA-MB-231 cells that were transfected with Lin28-si1 or Lin28-si2, the proportion of ALDH-positive cells was reduced when compared with transfection reagent treatment control cells (Figure 5E).

### Lin28 expression in breast cancer was correlated with tumor invasiveness

**Figure 6:**
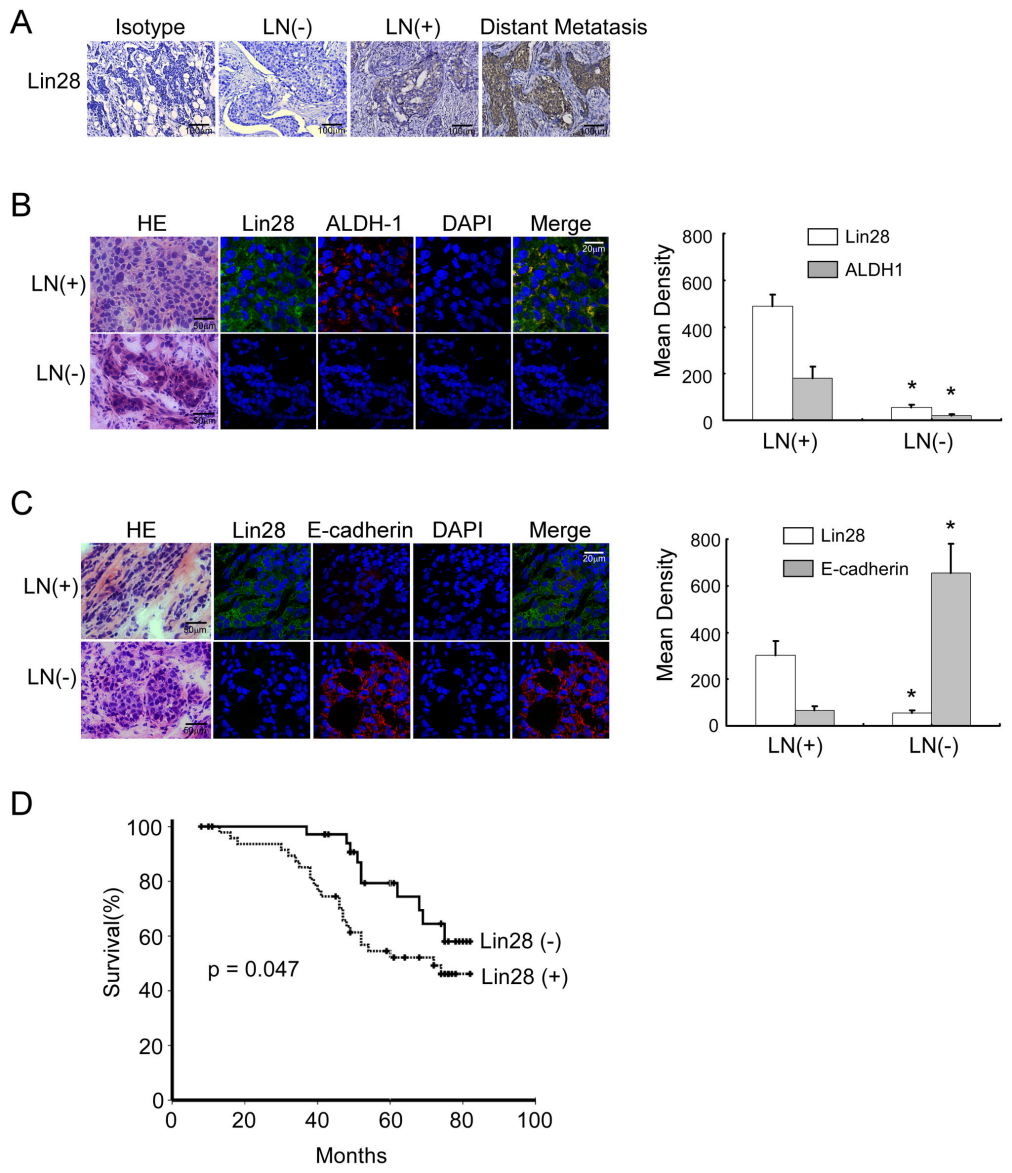
Lin28 expression in breast cancer is correlated with tumor invasiveness. A. Lin28 immunohistochemical staining was performed in samples obtained from breast cancer patients with or without axillary lymph node metastases or with distant metastases. An isotype-matched antibody was used as a negative control. B and C. (Left) Confocal microscopy for double immunostaining of Lin28 (green) and cytoplasmic expression of ALDH1 (red) (B) and membrane expression of E-cadherin (red) (C) in breast cancer tissue samples that had been obtained from patients with (upper) or without (lower) lymph node metastases. H&E staining was performed on frozen sections of the same breast cancer tissues. Cell nuclei (blue) were counterstained with DAPI. (Right) Statistical mean of the fluorescence density of Lin28, ALDH1 and E-cadherin in LN (+) and LN (-) breast cancer sections. D. A Kaplan-Meier survival curve was established for patients who had breast cancers with low and high Lin28 expression, with a median follow-up period of 57 months, p < 0.05.

To determine the correlation between Lin28 and metastasis in breast cancer, we examined Lin28 expression by immunohistochemistry in 86 primary breast cancer tissue samples. We found that the expression of Lin28 was low in breast cancers that had not metastasized to lymph nodes, whereas Lin28 expression was higher in breast cancers that had metastasized to lymph nodes. In addition, Lin28 expression was much higher in patients who had distant metastases (Figure 6A). An isotype-matched antibody was used as a negative control in breast cancers with distant metastases.

We then correlated Lin28-positive breast cancer cell counts with the clinicopathological status of the patients with breast cancer (Table 3). The number of Lin28-positive breast cancer cells markedly increased as tumor burden increased, which was deﬁned by tumor size (p < 0.01), staging (p < 0.01) and aggressive tumor biology using advanced histopathological grading (p < 0.01). Additionally, Lin28-positive breast cancer cells were more abundant in primary tumors that had axillary lymph node (p < 0.001) and distal metastases (p < 0.05) as well as PR positive breast tumors. However, our cohort did not reveal a signiﬁcant correlation between patient prognoses and ER or Her-2 expression.

**Table 3:** Correlation of Lin28 Expression with Clinicopathological Status in 86 Cases of Patients with Breast Cancer.

	Lin28(-)	Lin28(+)	*p* Value
Age			
<=45	20	23	
>45	15	28	0.106
Menopause	3	7	
Pre-menopausal	20	29	
Post-menopausal	11	15	0.02
Tumor Size (cm)			
<=2	24	16	
>2	11	35	<0.01
Stage			
Ⅰ	23	7	
Ⅱ	9	27	
Ⅲ	2	12	
Ⅳ	1	6	<0.01
Histological Grade			
Ⅰ	16	2	
Ⅱ	14	17	
Ⅲ	5	32	<0.01
Lymph Node Metastasis			
(-)	29	18	
(+)	6	33	<0.001
Distant Metastasis*			
(-)	33	36	
(+)	2	15	<0.05
ER			
(-)	14	17	
(+)	21	34	0.400
PR			
(-)	18	14	
(+)	17	37	0.024
Her-2			
(-)	16	35	
(+)	19	16	0.090

^*^ , Distant metastasis identiﬁed during postoperative follow-up.

To study the correlations between Lin28, Aldehyde dehydrogenase 1 (ALDH1), and E-cadherin expression in vivo, we performed double immunofluorescence staining in patient-derived, frozen primary breast cancer tissue samples. Overexpression of Lin28 and ALDH1 was found in breast cancer patients who had lymph node metastases (Figure 6B, upper panel), in which E-cadherin was downregulated (Figure 6C, upper panel). In breast cancer patients who did not have lymph node metastases, Lin28 and ALDH1 were expressed at low levels (Figure 6B, lower panel), whereas E-cadherin levels were elevated (Figure 6C, lower panel).

A Kaplan-Meier survival curve with a median follow-up period of 57 months demonstrated that patients with low Lin28 expression levels (n=35) survived signiﬁcantly longer than those with high Lin28 expression levels (n=51) (Figure 6D, p = 0.047). Taken together, these results demonstrated that Lin28 was associated with advanced disease and poor clinical outcomes in breast cancer.

## Discussion

Lin28 was determined to be a predictor of poor prognosis in a subset of human tumors [3]. In addition, Lin28 depletion suppressed bone metastases in mice bearing breast cancer cells [27]. Although Lin28 is known to promote malignancy by inhibiting the biogenesis of let-7, which functions as a tumor suppressor [28,29], the role of the Lin28/let-7 axis in the epithelial to mesenchymal transition in breast cancer remains unknown. In the present study, we found that Lin28 was highly expressed in mesenchymal (M)-type cells (MDA-MB-231 and SK-3rd), but it was barely detectable in epithelial (E)-type cells (MCF-7 and BT-474). However, Lin 28b did not have this expression pattern. By overexpressing and suppressing Lin28, we demonstrated that Lin28 significantly decreased the expression of E-cadherin and increased the expression of vimentin in breast cancer cells via let-7a repression, subsequently increasing the mammosphere formation rate and ALDH1 activity and promoting colony formation, adhesion and migration in breast cancer cells. Furthermore, Lin28 overexpression was found in metastatic breast cancers and was strongly predictive of poor prognosis in breast cancer patients.

EMT is known to facilitate tissue remodeling from the epithelial phenotype to the mesenchymal phenotype and is considered to be a prerequisite for tumor infiltration and metastasis [16–18]. A RNA binding protein, Sam68, was found to promote the malignant transformation of epithelial cancers by inducing the posttranscriptional regulation of EMT [30]. Another RNA binding protein, Rbfox2, is an essential regulator of EMT-driven alternative splicing and is a mediator of cellular invasion [31,32]. Depletion of Rbfox2 in mesenchymal cells elicited significant changes in cell morphology and motility and promoted conversion to an epithelial phenotype in breast cancer cells [33]. Lin28 is a highly conserved RNA binding protein [1,34], which has been reported to be up-regulated in breast cancer and is correlated with advanced tumor stage and poor prognosis [28]. When further confirming the post-transcriptional regulation of EMT by the RAN binding protein, we found that overexpression of Lin28 in breast cancer cells remarkably decreased the expression of the epithelial marker E-cadherin and increased the expression of the mesenchymal marker vimentin in addition to promoting significant changes in cell morphology toward a mesenchymal phenotype, suggesting that Lin28 induced the EMT in breast cancer cells.

Lin28 is known to bind to the terminal loops of the precursors of let-7 family members and selectively block their processing into mature miRNAs [11,34,35]. In this study, we detected the expression of all Let-7 family members in vec, pc-Lin28-1 and pc-Lin28-2 MCF-7 cells. Ectopic expression of Lin28 in pc-Lin28-1 and pc-Lin28-2 cells dramatically decreased the abundance of Let-7. Because Let-7a has a higher expression level in vec MCF-7 cells and larger regulation extent by Lin28, we focused our research interests on the Let-7a-mediated, Lin28-induced EMT. Meanwhile, our results showed that pc-Lin28-1 had a more significant inhibitory effect on Let-7 than pc-Lin28-2; therefore, we used pc-Lin28-1 as a Lin28 overexpression cellular model except where otherwise indicated. Consistent with the Taqman qPCR results, qPCR with SYBR probes also demonstrated that Lin28 overexpression in breast cancer cells led to let-7a downregulation, while silencing the overexpressed Lin28 increased the level of let-7a. To rule out the off-target effects of Lin28 siRNAs, we constructed a mutated Lin28 expression vector (Lin28-resmut) that contained 6 site mutations in the siRNAs targeting sequences that did not change the Lin28 amino acid sequence. Lin28-resmut totally reversed the effects of siRNAs, which means that the Lin28-resmut was insensitive to the Lin28 siRNAs and the recovery of Let-7 expression by siRNAs was due to Lin28 knockdown. A recent study reported that let-7 functions as a novel regulator of the epithelial to mesenchymal transition in oral cancer [16]. Knockdown of let-7 significantly promoted EMT traits, whereas overexpression of let-7 efficiently reversed the EMT phenotype in oral and pancreatic cancer cells [16,19]. Moreover, downregulation of let-7 levels initiated and maintained the oncostatin M-induced EMT via high-mobility group A protein 2 in breast cancer cells [20]. Our data demonstrated that the restored expression of let-7, either using let-7 mimics or by depleting Lin28, dramatically up-regulated E-cadherin expression and downregulated vimentin expression in Lin28 transfectants, suggesting that overexpression of let-7 led to the reversal of EMT. Hence, Lin28 facilitated the EMT via downregulation of let-7a and further promoted adhesion and migration in breast cancer cells. 

Lin28 is a reprogramming factor of adult human fibroblasts that has been described to induce pluripotent stem (iPS) cells [23]. It is reported that Lin28 facilitates the expression of the pivotal pluripotency factor Oct4 at the post-transcriptional level in human embryonic stem cells [24]. Cai et al reported that loss of function of Lin28 impairs Wnt-β-catenin-pathway-mediated let-7 inhibition and breast cancer stem cell expansion, and the enforced expression of let-7 blocks the Wnt-β-catenin pathway-stimulated breast cancer stem cell phenotype [36]. In our study, we found that forced expression of Lin28 in breast cancer cells markedly raised the rate of mammosphere formation and the ability of self-renewal as well as stemness marker ALDH1 activity. These findings indicated Lin28 could induce the stemness of breast cancer cells. Let-7 is an important downstream effector of Lin28, which had been reported to regulate self-renewal and tumorigenicity of breast cancer cells [22], and antagonizing let-7 using antisense oligonucleotide inhibitors enhances reprogramming of mouse ﬁbroblasts to iPSCs [37]. Because Lin28 represses let-7 expression [11,29,38,39], we speculated that Lin28a might mediate the stemness of breast cancer via downregulation of let-7.

It has been reported that the EMT and cancer stem cell (CSC) phenotypes have been independently linked with metastatic progression, drug resistance and disease recurrence [25,40]. Induction of the EMT in immortalized and transformed human mammary epithelial cells signiﬁcantly enhanced their self-renewal and tumor-initiating capabilities and led to the expression of stem-cell markers that are typically associated with the stemness of breast cancer cells [41]. In addition, chronic overexpression of the homeobox protein Six1 in the mouse mammary gland generated highly aggressive tumors with an EMT phenotype and stem cell features [42]. Our data demonstrated that high co-expression of Lin28 and ALDH1 was found in breast cancers with lymph node metastasis in which the expression of E-cadherin was low. These results provided the vital evidence for the emergence of cells with combined EMT/CSC phenotypes. Lin28 mediated downregulation of let-7a and subsequently induced EMT and stemness of breast cancer cells. Together with recent findings showing that let-7 was connected to EMT and stem cell formation [43], we confirmed that Lin28 was implicated in the molecular pathway linking EMT and CSC via let-7a.

Given the pivotal role of Lin28 in promoting EMT and stemness via inhibition of let-7a, targeting Lin28 may be expected to be a therapeutic strategy that can be used to eliminate metastatic cells to prevent recurrence and improve long-term survival of breast cancer patients.

## Supporting Information

Figure S1 **Lin28B expression in breast cancer cell lines.** Differential expression of Lin28 was analyzed by Western blotting in multiple breast cell lines, including MCF-7, SK-3rd, MCF-10A, MDA-MB-231 and BT-474.
(TIF)

Figure S2 **Expression of Let-7 miRNA family using qPCR assay with Taqman probes.** Quantitative RT-PCR for human Let-7 mRNA, normalized to U6 mRNA, in vec, pc-Lin28-1, pc-Lin28-2 MCF-7 cells.
(TIF)

Figure S3 **Construct of siRNA-insensitive Lin28 expression plasimd.** The expression plasmid encoding siRNA-insensitive Lin28 contains 3 site mutations within each siRNA targeting sequences.
(TIF)
