# Supplementary figures and images for: Lin28 Induces Epithelial-to-Mesenchymal Transition and Stemness via Downregulation of Let-7a in Breast Cancer Cells
Source: PLoS One. 2013 Dec 11;8(12):e83083. doi: 10.1371/journal.pone.0083083 (PMC3859647; doi:10.1371/journal.pone.0083083)

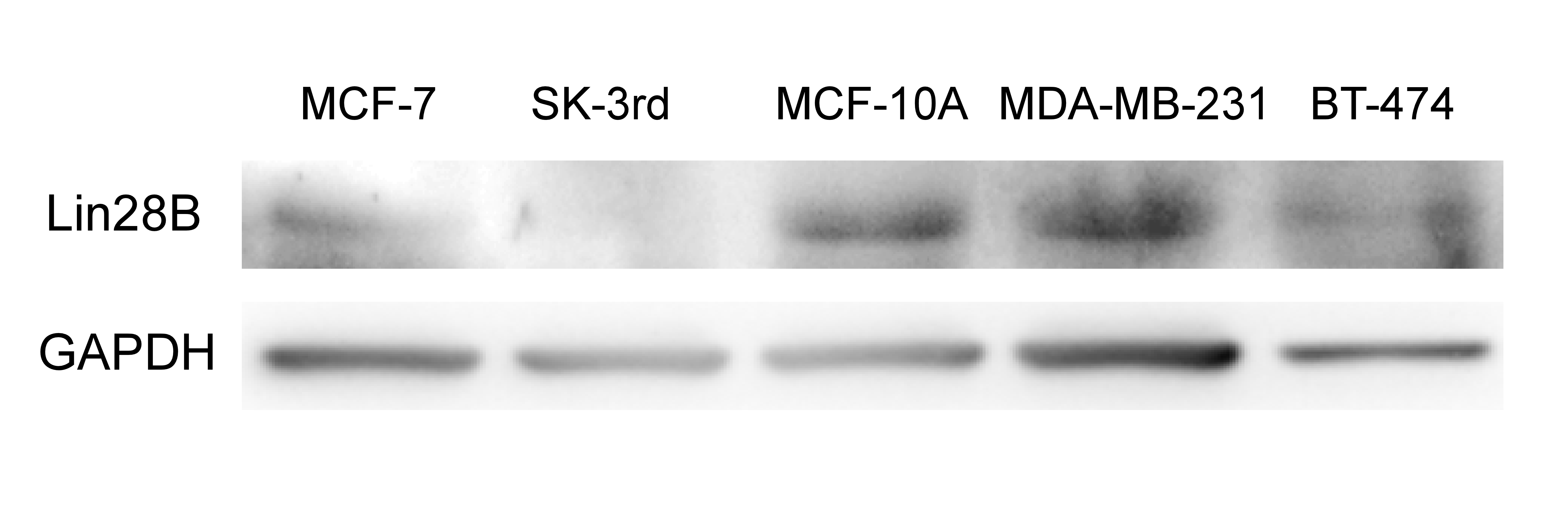

Supplement: Figure S1 — Lin28B expression in breast cancer cell lines. Differential expression of Lin28 was analyzed by Western blotting in multiple breast cell lines, including MCF-7, SK-3rd, MCF-10A, MDA-MB-231 and BT-474. (TIF) [file pone.0083083.s001.tif]

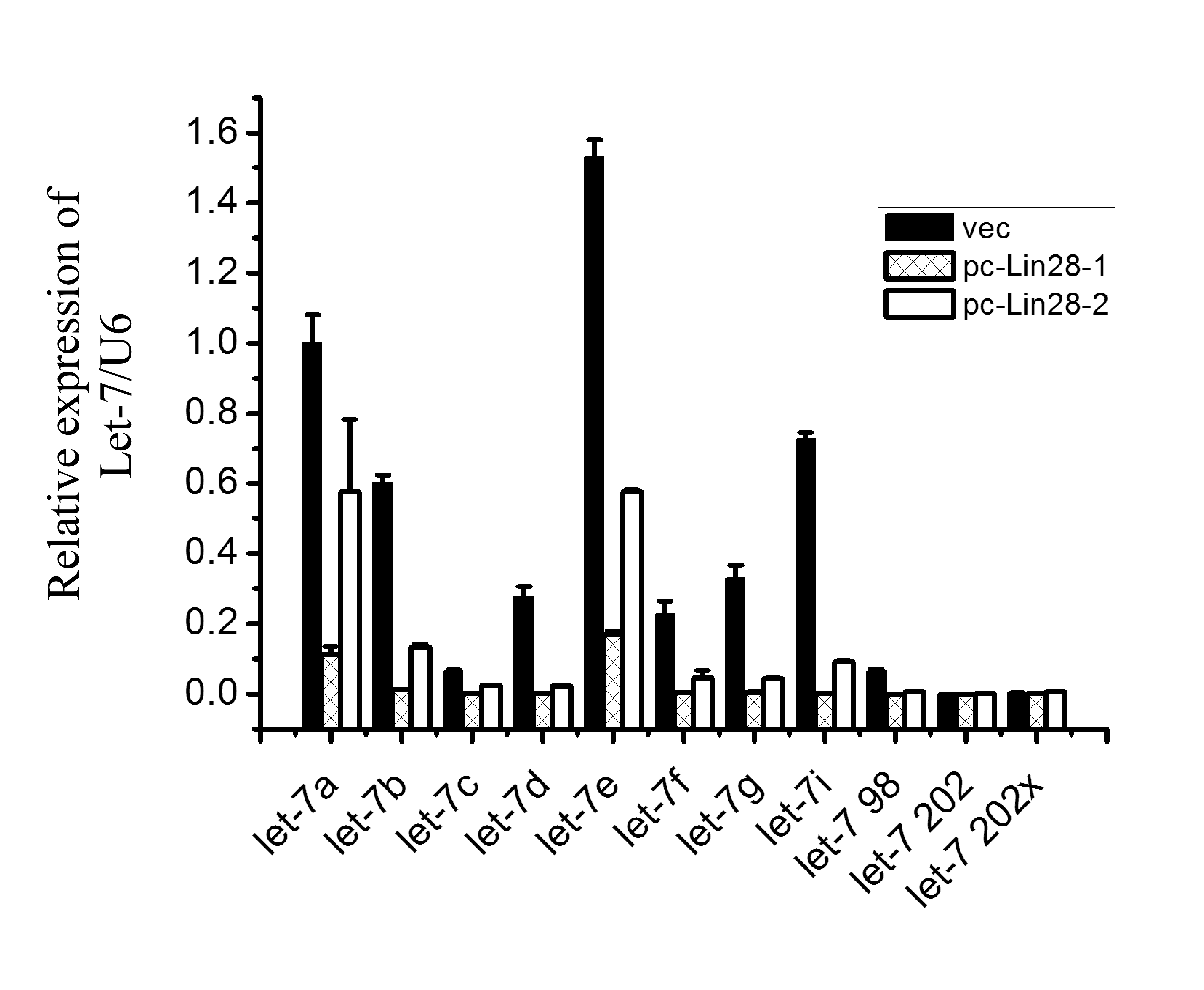

Supplement: Figure S2 — Expression of Let-7 miRNA family using qPCR assay with Taqman probes. Quantitative RT-PCR for human Let-7 mRNA, normalized to U6 mRNA, in vec, pc-Lin28-1, pc-Lin28-2 MCF-7 cells. (TIF) [file pone.0083083.s002.tif]
